# Probing the use of visual search as a tool to enhance reading speed

**DOI:** 10.3758/s13414-026-03336-2

**Published:** 2026-09-23

**Authors:** Francesco Carabba, Chiara Borsati, Giulia Fontana, Eric Altieri, Michele Vicovaro, Luca Battaglini

**Affiliations:** 1 Cognitive and Educational Sciences Lab (CESLab), Faculty of Education, Free University of Bolzano-Bozen, 39042 Bressanone-Brixen, BZ Italy; 2 Department of General Psychology, University of Padova, Via Venezia, 8, 35131 Padova, Italy; 3 Centro di Ateneo dei Servizi Clinici Universitari Psicologici (SCUP), University of Padova, Via Tommaseo, 47/A, 35131 Padova, Italy; 4 NeuroVis.U.S. Laboratory, University of Padova, Via Tommaseo, 47/A, 35131 Padova, Italy

**Keywords:** Reading, Visual search, Training, Perceptual learning, Magnocellular-Dorsal system

## Abstract

**Abstract:**

The reading mechanism is associated with perceptual, attentional, and oculomotor processes that partly overlap with functions attributed to the magnocellular-dorsal system. On this basis, we examined whether a home-based visual-search training program, without phonological, orthographic, or reading components, could influence reading-related performance in adult typical readers. Participants were assigned to one of three training versions that differed in the visual features of Gabor patterns to be detected (orientation, phase, or configuration), or to a no-training control group. Training performance indicated that the three visual-search versions differed in difficulty: The orientation task was relatively easy, the phase task was the most perceptually demanding, and the configuration task was intermediate. Regression analyses controlling for pretest performance showed lower adjusted posttest reading times, relative to the no-training group, for the phase group in word reading and lexical decision, and for the configuration group in nonword reading. No consistent training-related advantage was observed for accuracy. These results suggest that demanding visual-search training may be associated with changes in some timed reading-related outcomes, but the effects were outcome-specific and should be interpreted as preliminary. Further work is needed to disentangle task difficulty, search mode, and training dose, and to determine whether similar effects generalize to children or to readers with dyslexia.

**Open practices statement:**

The raw trial-level data for all experimental conditions are publicly available in the OSF repository (https://doi.org/10.17605/OSF.IO/XTY4S). The repository also contains the code for the analyses described in the Sensitivity Analysis and Type I Error Rate section. The experiment and analyses were not preregistered.

**Supplementary Information:**

The online version contains supplementary material available at https://doi.org/10.3758/s13414-026-03336-2.

## Introduction

Reading is a complex and multicomponent task that involves basic visuoperceptive, oculomotor, and attentional skills, together with access to symbolic orthographic and lexico-semantic knowledge and the activation of phonological representations (Danelli et al., 2012). The previous generation of reading models dates back to around the 1980 s and 1990 s and aims at providing a framework of lexical structure and processing (Frost, 2012), with theories primarily concerning the phonological domain. Subsequent computational models of reading have mostly focused on the initial phase of visual word recognition and have introduced various forms of context-sensitive coding (Frost, 2012). These theories explain not only how we read but also reading problems such as dyslexia, a specific learning disorder (see Supplementary Material 1 for more details).

In this study, we examine the magnocellular hypothesis (Livingstone et al., 1991; Stein & Walsh, 1997), which proposes that impaired development and function of the magnocellular-dorsal system may represent a significant contributing factor—or possibly a consequence—in the etiology of dyslexia. This neural pathway comprises magnocellular neurons that excel in rapid temporal processing (Stein, 2019) and form the foundation of the occipito-parietal route, commonly referred to as the "where" pathway due to its specialization in processing visual-spatial information. Research suggests that the magnocellular-dorsal system plays a crucial role in modulating the distribution of attentional resources during reading. Specifically, spatial attention mechanisms facilitated by this pathway appear fundamental to the development of reading skills, enabling the processing of letters at various positions within text, facilitating efficient orthographic-to-phonological code conversion, and supporting rapid semantic access from visual input (Grainger et al., 2016). In addition, some studies have shown a relationship between difficulties concerning visuo-spatial attention and difficulties in reading (Franceschini et al., 2012; Iles et al., 2000; Nguyen et al., 2021). Furthermore, eye movements play a key role in reading and dyslexia. For example, in dyslexia, control over eye muscles is less focused over time and less able to stabilize the eyes during the binocular fixation required for reading (Stein, 2001; Stein & Walsh, 1997). The magnocellular theory has faced challenges over time (Ciavarelli et al., 2021; Contemori et al., 2019). Some authors suggest that it is not possible to reduce the etiology of dyslexia to a single cause (Edwards & Schatschneider, 2020; Skottun & Skoyles, 2006). However, ample empirical evidence supports involvement of perceptual and visuo-spatial skills in reading.

On this basis, we hypothesized that visual training, focused on exercising these abilities, could promote perceptual learning (PL) with positive outcomes in reading tasks. PL refers to the phenomenon that practice in perceptual tasks often substantially improves perceptual performance (Lu et al., 2011). It is a manifestation of cortical plasticity and results in a change that appears to have the potential to persist over time. In particular, visual perceptual learning (VPL) denotes a long-term improvement in visual task performance as a result of visual experience (Maniglia & Seitz, 2018; Reavis et al., 2016; Sasaki et al., 2010). Positive outcomes of VPL were shown in different clinical populations such as hemianopia (Casco et al., 2018), amblyopia (Barollo et al., 2017), macular degeneration (Maniglia et al., 2016) and also in dyslexic readers (Gori et al., 2016). In this work, the implementation of a visual search training program is grounded in the strong link between visual search and visuo-spatial skills, as well as aspects involved in the magnocellular-dorsal system (visual attention and relative shifting, eye movements, temporal processing). Visual search can be defined as the act of visually exploring space to efficiently detect a specific element of interest, with attention being a crucial component. Attention is influenced by various factors, including bottom-up and top-down ones (Bravo & Nakayama, 1992; Wolfe & Horowitz, 2017). Typically, performance concerning visual search shows large effects from training (Czerwinski et al., 1992).

There are currently different types of training that have been designed and tested for reading ability. Some previous studies have highlighted the effects of exercise programs related to the activity of the magnocellular-dorsal system. Chouake et al. (2012) examined whether repeated use of the magnocellular stream, through a coherent motion detection task, could improve reading by strengthening relevant neural pathways. Similarly, Lawton (2011) reported improvements following direction discrimination training with sine-wave gratings, and Peters et al. (2019) reviewed evidence for visual perceptual training based on low-level visual-attentional mechanisms. Caldani et al. (2020) used a brief training battery that combined oculomotor exercises with visual-search tasks. Specifically, they reported that a single approximately 10-min session combining oculomotor exercises (horizontal visually guided saccades and pursuit tasks) with visual-search tasks was followed by faster reading and shorter fixation durations in French children with dyslexia or reading disorders. This finding is highly relevant for the link between visual-attentional training and reading, but the design does not allow the specific contribution of the visual-search component to be isolated from that of the oculomotor exercises.

To the best of our knowledge, the study by Caldani et al. (2020) therefore represents the closest previous attempt to use visual-search training in relation to reading ability, but the inclusion of oculomotor exercises constitutes an important difference from the present approach. By implementing a visual-search intervention restricted to a single task family, our design was intended to reduce ambiguity regarding which component of training might drive any observed reading-related changes. Even other studies linking visual search, reading, and dyslexia, such as those involving action video games (Franceschini et al., 2015), tend to engage multiple components and sensory systems. Here, we propose a home-based visual-search training that excludes phonological, orthographic, or reading elements during practice, while engaging visual selection, visuo-spatial attention, and related processes.

**Fig. 1:**
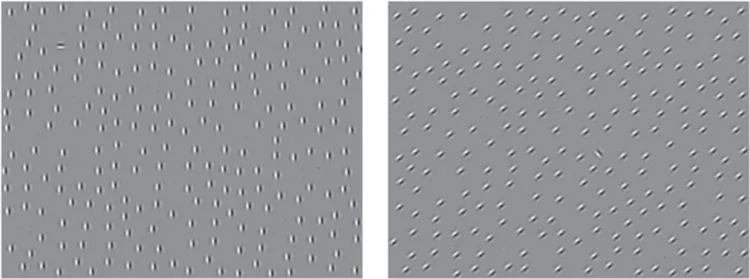
Example of visual search in “orientation” version. Left: target stimulus present in the upper left corner. Right: target stimulus present close to the center

**Fig. 2:**
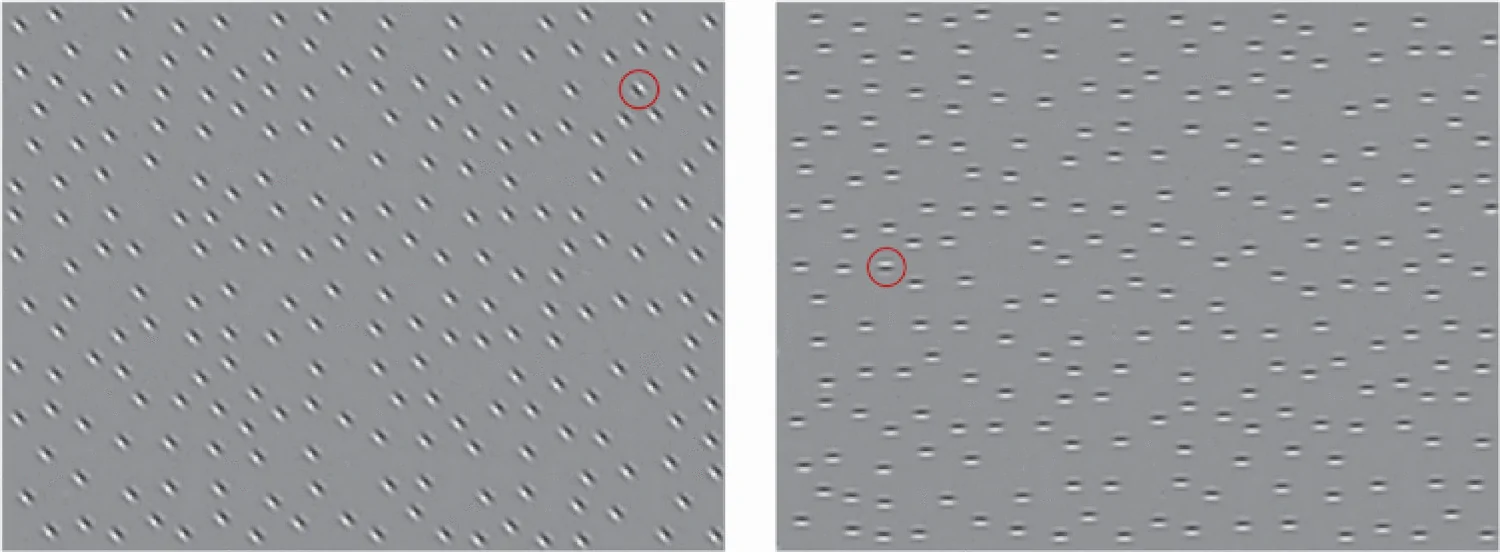
Example of visual search in “phase” version. Left: target stimulus present in the upper right corner. Right: target stimulus present in the center left

**Fig. 3:**
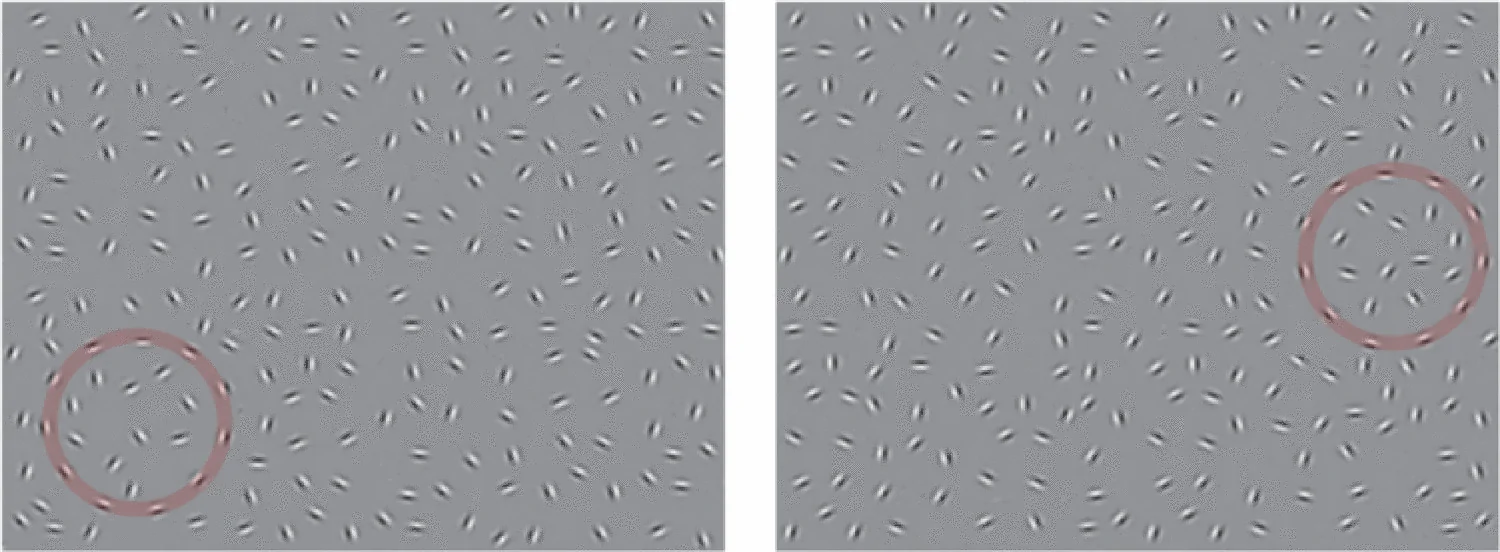
Example of visual search in “configuration” version. Left: target stimulus present in the lower left corner. Right: target stimulus present in the center right

Our primary goal is to investigate whether visual search training alone can induce changes in reading ability even among typical reader participants. Furthermore, we explored different visual search setups to evaluate if they differed in their effectiveness at producing changes, and to what degree. We provided three distinct versions of the task. All the stimuli were Gabor patches. The first visual search task version, called "orientation," involved a single-stimulus target with a different orientation (Fig. 1) compared with all distractor stimuli. This manipulation was designed to examine whether improvements in reading can be obtained by training visual discrimination of simple orientation features. A second version, named "phase," involved a single-stimulus target with a different phase (a reversal of the black and white colors that characterize the stimulus) compared with the other distractors (Fig. 2), resulting in a highly camouflaged target. This phase manipulation was specifically designed to examine how visual discrimination based solely on luminance reversal patterns—a process requiring detailed scrutiny of individual stimuli without involving contour integration—might influence reading processes. A third task version, called "configuration," involved a target consisting of multiple Gabor patches arranged in a circular configuration, thus requiring visual grouping abilities for detection (Fig. 3). This configuration was proposed following a study by Szwed et al. (2012) that demonstrated enhancement of early visual contour integration processes due to reading acquisition. Illiterate people seem to have greater difficulty in grouping Gabor patches circularly arranged in a single percept (Szwed et al., 2012). This suggests that reading experience may enhance neural mechanisms supporting contour integration. We hypothesized that the relationship might be bidirectional—if reading enhances contour integration abilities, then specifically training contour integration might conversely benefit reading performance even in typical readers who have already developed these skills to some degree. This potential link between feature integration and reading led us to ask whether a demanding visual-search task with single-Gabor targets would be sufficient to affect reading-related performance, or whether a task requiring contour integration would produce stronger effects. We also sought to examine these aspects in typical readers before testing special populations. We chose to begin with typical readers as this approach allows us to establish baseline effects of visual search training on reading in the neurotypical brain, providing a critical foundation for understanding the potential of this intervention. If successful with typical readers, this would suggest fundamental processes linking visual search skills and reading abilities that transcend reading proficiency levels. Subsequently, this training might be proposed to dyslexic readers, although we recognize that brain structures and connectivity may differ in this population (Finn et al., 2014), potentially requiring adaptations to the protocol. Establishing efficacy in typical readers would provide the necessary empirical justification for clinical trials with dyslexic populations, where the potential benefits might be even more pronounced due to their specific deficits in magnocellular-dorsal processing.

## Material and methods

### Participants

A total of 85 adult typical reader participants, 52 women (*M*_age_ = 31.62 years, *SD* = 14.80) and 33 men (*M*_age_ = 37.73 years, *SD* = 17.73), took part in this study. The experimental project was conducted in accordance with the Declaration of Helsinki and approved by the Ethics Committee of Psychological Research at the University of Padua (Protocol No. 4485). Written informed consent for participation and processing of personal data was obtained at the beginning of the study. Participants were native Italian speakers. They had normal visual acuity or had corrected their vision with glasses or contact lenses as necessary. The reading tasks were administered in Italian. Participants were classified as typical readers on the basis of no self-reported history of dyslexia, reading disorder, language disorder, neurological disorder, or uncorrected visual impairment. Thus, the term "typical readers" refers to adults without reported reading difficulties rather than to participants who underwent a full diagnostic assessment of reading ability. Because Italian has a relatively transparent orthography, the present findings should be interpreted with caution when considering their generalizability to languages with less transparent orthographic systems.

The sample size was constrained by time and financial resources; therefore, no a priori power analysis was conducted. Instead, a sensitivity analysis was performed to determine, given the available sample size, the minimum adjusted group differences at posttest associated with a statistical power of .80 in the regression framework. A more detailed description of this analysis and its results is provided in the Sensitivity Analysis and Type I Error Rate section. In brief, the results indicated that the study was sufficiently powered to detect only relatively large training effects, and that the Type I error rate was lower than the conventional α level of .05 in most cases, reinforcing the exploratory nature of the present findings. However, for the dependent variables Errors in Test 3 and Errors in DLC, the simulations showed that no plausible posttest mean difference yielded adequate power.

### Apparatus

The reading tests were conducted using Test 2 and Test 3 extracted from the Dyslexia and Developmental Dysorthographia Evaluation Battery (DDE-2; Sartori et al., 2007) and the lexical decision test for assessing reading skills DLC (Caldarola et al., 2012). The computerized visual search training required MATLAB Psychtoolbox (Brainard, 1997; Pelli, 1997) and MATLAB Runtime R2017b (9.3) and was run on participants’ laptops.

### Tests and stimuli

#### Reading tests

Reading proficiency was assessed using Test 2 and Test 3 from DDE-2 (Sartori et al., 2007). The DDE-2 test–retest reliability coefficients are .77 for speed and .56 for accuracy; additionally, control groups were useful for checking a possible practice effect. Hunsley and Mash (2018) suggest that test–retest reliability is “adequate” when correlations are at least.70 over days or weeks. Since the DDE-2 was retested after 20 days, the speed component can be considered at least adequately reliable. Test 2 is a words reading test that requires participants to read lists of words aloud as quickly as possible and without committing errors. Test 3 is a nonwords reading test and requires participants to read lists of nonwords (sequences of meaningless letters) aloud quickly and without errors. Because Italian has a relatively transparent orthography, nonwords can be pronounced by applying standard grapheme-to-phoneme conversion rules. The lexical decision test for assessing reading skills DLC (Caldarola et al., 2012) comprises a list of words and nonwords. The words were Italian and were either short (range: 4–5 letters, average length: 4.50) or long (range: 8–9 letters, average length: 8.17). The nonwords were obtained by changing one or two letters in words of the same length. The test includes 60 words with high frequency of use and familiarity and 60 nonwords, entered in random order. Because the words are familiar, any misidentification of a word as a nonword is more likely to reflect misreading than lack of familiarity. The test requires participants to read silently and mark the nonwords. The evaluation of performance is based on reading time and correct responses (adequate recognition of nonwords). The overarching goal was to utilize brief reading assessments that provide simple and prompt indicators of reading proficiency.

Reading speed was indexed by total completion time. For DDE-2 Test 2 and Test 3, timing started when the participant began reading the first item aloud and stopped when the participant finished reading the last item. Errors were recorded separately, and no error-based penalty or correction was applied to the time score. For the DLC lexical decision task, the time score reflected the total time required to complete the silent lexical decision task, whereas accuracy was scored separately. Thus, speed and accuracy were treated as distinct dependent variables.

#### Visual search training

The visual search training was implemented using Gabor patch stimuli, products of a sine wave luminance grating and a circular Gaussian envelope (Equation 1). The carrier spatial frequency of the Gabor patches was about 5 c/deg and their contrast was 95% (Michelson Contrast). Small variations in these parameters might be due to the utilization of different screens. The training consisted of 9 consecutive days with seven blocks of exercise performed daily. Each block comprised 144 images (half containing the target stimulus and the other half not containing it) presented one after the other at the center of the computer screen with over 100 Gabor stimuli, including the potential target and distractors. The image remained static until a response was given or for a maximum of 10 s. In detail, three distinct training versions were implemented, each characterizing a different group of participants.

1 $$G\left(x,y\right)=\mathit{cos}\left(\frac{2\pi }{\lambda }X+\varphi \right){e}^{\left(\frac{{x}^{2}+{y}^{2}}{{\sigma }^{2}}\right)}$$

#### Visual search training “orientation” version

In this version, the visual search task involved co-oriented Gabor stimuli with varying orientations across trials that were 0° (vertical), 45° (oblique), 90° (horizontal), and 135° (oblique). The target was a single Gabor with an orthogonal orientation (Fig. 1). This type of training featured an easy-to-find target that almost stood out independently within the visual search pattern.

#### Visual search training “phase” version

In this version, the visual search task involved co-oriented Gabor stimuli with varying orientations across trials (0°, 45°, 90°, and 135°). The target was a single Gabor with an inverted phase (a reversal of the black and white colors that characterize the stimulus) relative to all other distractors. This manipulation substantially increased task difficulty (Fig. 2).

#### Visual search training “configuration” version

The target, this time, was not represented by a single Gabor but rather by a series of Gabor patches arranged to form a circular configuration presented with many other Gabor distractors with random orientation (Fig. 3). This type of training incorporated a target with grouping features, specifically implemented based on a study conducted by Szwed et al. (2012) regarding the improvement of the early visual process of contour integration due to reading acquisition (see also Gerván & Kovács, 2010; Kovács & Julesz, 1993; Kozma-Wiebe et al., 2006).

#### No-training group

We also included, as an additional control condition, a group of participants who underwent only pretest and posttest for reading skills without any training during the interim period.

### Procedure

Of the 85 participants recruited, 42 were randomly assigned to one of the three training groups (14 participants per training group) and 43 did not undergo any training (control group). The decision to have a larger control group than experimental groups was dictated by feasibility constraints: the experimental trainings are highly demanding for participants (and resource-intensive to administer), and we did not have sufficient time and resources to recruit and test additional participants in those conditions. Importantly, increasing the size of the control group still provides several methodological advantages. First, it yields a more precise estimate of the no-training trajectory (i.e., practice effects, test–retest changes, and measurement noise), which is critical for interpreting any pre–post changes observed in the experimental groups. Indeed, a larger control group reduces the standard error of the control means, which improves the precision of between-group contrasts and effect size estimates that use the control group as the primary reference. Second, it increases power for detecting differences between each experimental group and the control group, which are the central comparisons in our design, even if the experimental group sizes remain unchanged. Finally, a larger control sample strengthens checks of baseline equivalence.

The study procedure consisted of pretest, training, and posttest. In the pretest and posttest, participants underwent reading tests (Test 2 and Test 3 from DDE-2 battery and the lexical decision test DLC). The experimenter recorded the time taken to read and noted any errors. Time recording, using a stopwatch, began at the start of the first word read and ended as soon as the last word read was finished. The three columns of words (or nonwords) were considered a single reading block. Pretest allowed for the definition of a baseline for each participant’s speed and accuracy. Then, the 9-day training phase began, with seven daily blocks of exercise based on a classic visual search paradigm, in which participants indicate whether a target is present or absent among distractors. The selection of a 9-day duration is informed by the standard timeframe for visual perceptual learning trainings (some examples of similar duration: An et al., 2012; Haris et al., 2020; He et al., 2023). Additionally, we conducted a pilot study indicating that this parameter was reasonable for achieving learning objectives and maintaining participant engagement. Participants, given written instructions and experiment-specific software, were tasked with detecting the presence or absence of the target for each picture (144 for each block) by pressing the appropriate button on a keyboard. The image remained fixed on the screen until a response was given or for a total time of 10 s after which, if neither key had been pressed, an “answer not given” was recorded (these trials were coded as 101 in the dataset and excluded from subsequent response times analysis). In each block the average response time, average accuracy and number of unanswered trials were recorded, as well as the RT and the specific response given for every single trial performed. The daily training duration varied by task (approximately 30 min for the "orientation," 90 min for the "phase," and 40 min for the "configuration" version). The posttest, conducted on the day following the conclusion of the training, replicated pretest procedures to enable pre-post comparisons and detect any training-related changes in reading performance.

## Results

### Visual search training

This was a home-based training. Most of the data were successfully retrieved, but technical issues resulted in the loss of 1.16% of participants’ responses (see Supplementary Material 2 for details and see https://doi.org/10.17605/OSF.IO/XTY4S for raw data).

### *D* prime

**Fig. 4:**
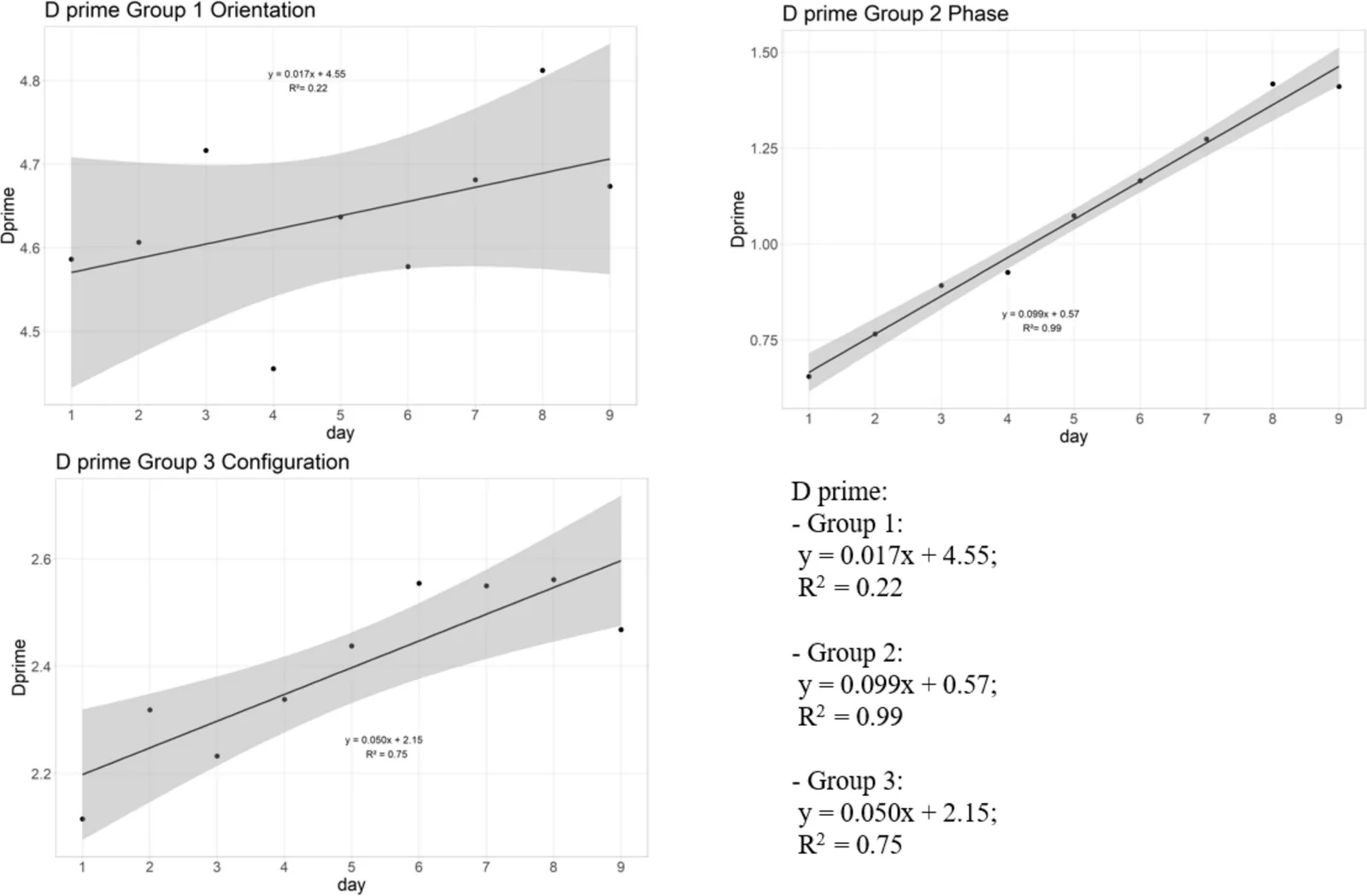
*D* prime linear regression model in the three groups. The data represent the *d′* daily mean of participants, the gray area shows the confidence interval. Top left: G1 has the highest *d′* values (very simple task). Top right: G2 has the lowest *d′* values (difficult and challenging task). Bottom left: G3 has intermediate *d′* values (intermediate task difficulty). Bottom right: linear regression model equation and *R*^2^ value in each of the three groups. G2 and G3 show greater positive slopes

The* d* prime (*d′*) index reflects the observer’s sensitivity and ability to discriminate signal from noise. Although mean RT is a common dependent variable in visual-search research, we also focused on sensitivity (*d′*) because our task included both target-present and target-absent trials and training could plausibly induce shifts in response criterion (e.g., a more liberal vs. conservative tendency to report target presence). Reporting *d′* allowed us to index discriminability while minimizing confounding by response bias. In addition, learning can involve speed–accuracy trade-offs; therefore, considering *d′* alongside RT provides a more complete characterization of training-related changes, distinguishing improvements in efficiency (faster responding) from improvements in perceptual/decisional sensitivity. In perceptual learning (PL), a positive slope of the line interpolating the *d′* values corresponding to the days of exercise is expected: values increase as the days pass. Therefore, *d′* is useful for assessing training engagement and improvement. A two-way mixed-model ANOVA was conducted. The sphericity assumption was assessed using Mauchly’s test, and when violated, the Greenhouse–Geisser correction was applied. Results showed a significant effect of Day factor, *F*(3.001,117.037) = 8.833, *p* < 0.001, η_p_^2^ = 0.185, indicating that, in general, participants performed less effectively on the first day of training and improved on the final day of training, suggesting perceptual learning. The Group factor was also significant, *F*(2,39) =174.202, *p* < 0.001, η_p_^2^ = 0.899, suggesting that at least one group was different from the others. The Day × Group interaction was not significant. Bonferroni-corrected post hoc *t* test confirmed a significant difference among all groups: “phase” vs. “orientation,” “configuration” vs. “orientation,” “phase” vs. “configuration” (Table 1). Furthermore, *d′* means in the groups reflected variations in task difficulty: “orientation” was very simple, “phase” difficult and challenging, and “configuration” occupied a middle ground between the two (Table 2 and Fig. 4). Individual daily *d′* trajectories for the three training groups are shown in Fig. S11 in Supplementary Material 2. We also examined the response criterion as an exploratory analysis. The corresponding results and descriptive statistics are reported in Tables S1 and S2, and individual daily trajectories are shown in Fig. S1, in Supplementary Material 2.

**Table 1:** *D* prime mean in the three groups and *p* value in post hoc comparisons among the groups

	Mean G1	Mean G2	Mean G3
	4.638375	1.064258	2.397497
	*SD* G1	*SD* G2	*SD* G3
	0.787555	0.530952	0.482429
	*p*_corr_	*t* value	Cohen’s *d*
G1 vs. G2	*p* < 0.001	16.640	6.289
G1 vs. G3	*p* < 0.001	10.769	4.070
G2 vs. G3	*p* < 0.001	−8.805	−3.328

G1 = “orientation” group; G2 = “phase” group; G3 = “configuration” group

**Table 2:** *D* prime means during the 9 days of training in the three groups

	*Day 1*	*Day 2*	*Day 3*	*Day 4*	*Day 5*	*Day 6*	*Day 7*	*Day 8*	*Day 9*
*Orientation*	4.586216	4.606381	4.71644	4.455044	4.636921	4.577437	4.681289	4.812197	4.673451
*Phase*	0.654828	0.76573	0.891937	0.925981	1.073647	1.164932	1.273645	1.417318	1.410306
*Configuration*	2.115378	2.318818	2.232687	2.33827	2.437798	2.554783	2.549931	2.561503	2.468307

#### Response times

**Fig. 5:**
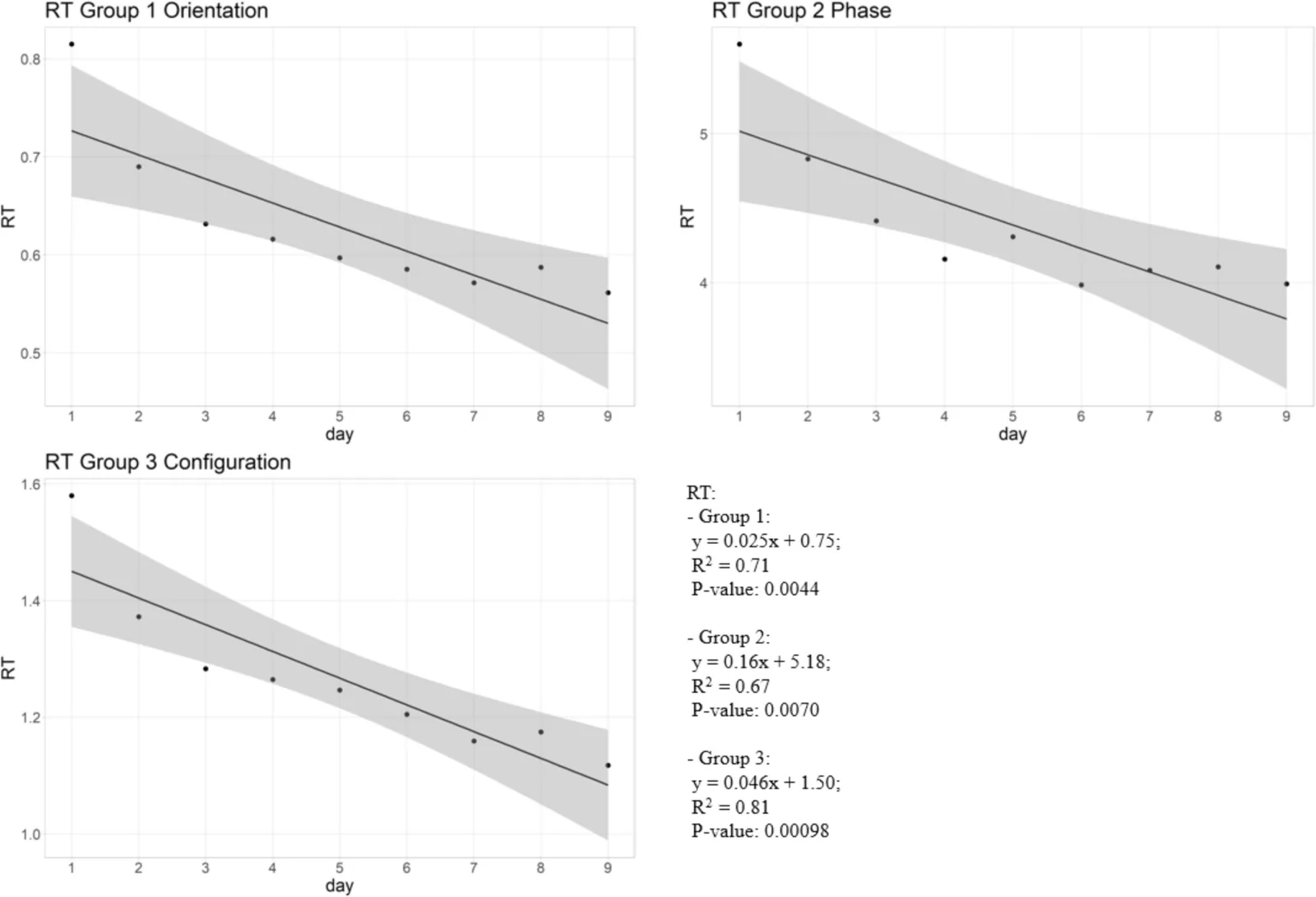
Response times linear regression model in the three groups. The data represent the response times daily mean of participants; the gray area shows the confidence interval. Top left: G1 has faster response times (simple task). Top right: G2 has slower RTs (difficult task). Bottom left: G3 has intermediate response times (intermediate task difficulty); Bottom right: linear regression model equation and *R*^*2*^ value in each of the three groups. G2 shows a greater slope of the line compared with G1 and G3 (greater improvement)

Response times were analyzed. A two-way mixed-model ANOVA showed a significant Day factor effect, *F*(2.613,101.925) = 21.401, *p* < 0.001, η_p_^2^ = 0.354, and a significant Group factor effect, *F*(2,39) = 104.849, *p* < 0.001, η_p_^2^ = 0.843. In general, participants became faster (response times decreased) as training days passed. Additionally, there were differences among the groups. Post hoc comparisons showed a significant difference among all groups: “phase” vs. “orientation,” “configuration” vs. “orientation,” “phase” vs. “configuration” (Table 3). The Day × Group interaction was also significant, *F*(5.227,101.925) = 6.971, *p* < 0.001, η_p_^2^ = 0.263. When examined by group, “phase” participants showed a greater slope of the line calculated for response times compared with the other two groups, resulting in the greatest improvement. The calculated means also indicate that response times in the “orientation” group were very fast (easy task), in the “phase” group slower (difficult task) and in the “configuration” group intermediate (Table 4 and Fig. 5). Individual daily response-time trajectories for the three training groups are shown in Fig. S12 in Supplementary Material 2. This reinforces the evidence that there is a difference in training difficulty among the groups. Furthermore, the number of timeouts also confirms this. Specifically, the mean number of time outs was higher in the “phase” group than in the other two groups, and higher in the “configuration” group than in the “orientation” group. Table S4 in the Supplementary Material 2 shows the average number of time outs for the different groups.

**Table 3:** Mean response times in the three groups and *p* value in post hoc comparisons among the groups

	Mean G1	Mean G2	Mean G3
	0.628412	4.387211	1.267122
	*SD* G1	*SD* G2	*SD* G3
	0.190895	1.39365	0.332152
	*p*_corr_	*t* value	Cohen’s *d*
**G1 vs. G2**	*p* < 0.001	−11.342	−4.287
**G1 vs. G3**	*p* < 0.001	−7.331	−2.771
**G2 vs. G3**	*p* < 0.001	9.238	3.492

G1 = “orientation” group; G2 = “phase” group; G3 = “configuration” group

**Table 4:** Mean response times during the 9 days of training in the three groups

	*Day 1*	*Day 2*	*Day 3*	*Day 4*	*Day 5*	*Day 6*	*Day 7*	*Day 8*	*Day 9*
*Orientation*	0.814963	0.689984	0.631787	0.616026	0.596983	0.585329	0.571693	0.587354	0.561593
*Phase*	5.602551	4.831708	4.415873	4.158123	4.309097	3.984993	4.083049	4.106654	3.992849
*Configuration*	1.579776	1.372283	1.283284	1.264792	1.246693	1.205063	1.159397	1.175025	1.117787

### Visual search training and reading ability

To examine potential effects on reading, we compared the training groups with a control group that completed only the pretest and posttests (i.e., no training). Table S5 in Supplementary Material 2 reports the descriptive statistics for each experimental condition and each dependent variable. Figs. S2–S7 in Supplementary Material 2 display the mean score and the individual observations for each dependent variable across conditions. To evaluate whether mean changes reflected consistent improvements across participants or were driven by a subset of individuals, we also inspected individual pre–post trajectories for each reading speed outcome. These plots are reported in Fig. S8–S10 in Supplementary Material 2. In addition, Tables S6 and S7 report individual change-score summaries for reading-time outcomes and for reading-accuracy outcomes. Because the no-training group completed the same reading assessments at both pretest and posttest, some improvement in reading speed was expected even in the absence of training. This change is likely attributable to test–retest/practice effects, including increased familiarity with the task format, reduced uncertainty about the testing procedure, and repeated exposure to the assessment context. For this reason, improvements observed in the training groups were not interpreted in isolation, but were evaluated relative to the no-training group while controlling for baseline performance. Posttest reading times and posttest reading errors were analyzed in separate linear regression models, with Group as the predictor (with the no-training group as the reference level) and the corresponding pretest score as a covariate. For each dependent variable, *p* values for the three planned comparisons between each training group and the no-training group were adjusted using the Bonferroni method. These analyses were conducted to test whether adjusted posttest group differences were present after controlling for baseline performance. The full results are presented in Table 5. Because power simulations showed that no plausible posttest mean difference could achieve adequate power for Test 3 and DLC error scores (see Sensitivity Analysis and Type I Error Rate section), we performed inferential analyses only on Test 2 errors. For Errors in Test 3 and DLC, only descriptive statistics are reported, and these are presented in the Supplementary Material (See Table S5 and Figs. S6–S7). This decision was motivated by the fact that, if the power simulations indicate that no plausible effect size for these outcomes can achieve adequate power, then testing for statistically significant differences between the experimental groups and the control group is not informative.

**Table 5:** Results of the linear models for reading times in Test 2, Test 3, and DLC and for reading errors in Test 2

Dependent variable	Effect	*b*	*β*	*SE*	*t* value (*df* = 80)	*p* value
Reading times Test 2	Pretest	0.79	0.86	0.05	17.57	<.001
	Orientation–no training	1.46	0.18	1.07	1.36	.532
	Configuration–no training	−2.18	−0.27	1.08	−2.01	.142
	Phase–no training	−4.06	−0.51	1.06	−3.83	<.001
Reading times Test 3	Pretest	0.80	0.92	0.04	17.93	<.001
	Orientation–no training	−1.37	−0.18	1.07	−1.28	.617
	Configuration–no training	−3.51	−0.46	1.11	−3.17	.007
	Phase–no training	−1.86	−0.24	1.07	−1.74	.260
Reading times DLC	Pretest	0.54	0.75	0.05	9.92	<.001
	Orientation–no training	−2.74	−0.16	3.59	−0.76	1
	Configuration–no training	−5.09	−0.30	3.65	−1.39	.501
	Phase–no training	−8.81	−0.51	3.59	−2.45	.049
Reading errors Test 2	Pretest	0.57	0.70	0.07	8.697	<.001
	Orientation–no training	0.11	0.12	0.20	0.55	1
	Configuration–no training	−0.25	−0.28	0.20	−1.25	.641
	Phase–no training	0.03	0.03	0.20	0.13	1

For each model, the unstandardized coefficient (*b*), standardized coefficient (*β*), standard error (*SE*), *t* value, and *p* value (Bonferroni corrected in the case of group comparisons) are reported

#### Reading time

In all reading-time models (Test 2, Test 3, and DLC), pretest reading times were a significant positive predictor of posttest reading times, Test 2: *b* = 0.79, *SE* = 0.05, *t*(80) = 17.57, *p* <.001; Test 3: *b* = 0.80, *SE* = 0.04, *t*(80) = 17.93, *p* <.001; DLC: *b* = 0.54, *SE* = 0.05, *t*(80) = 9.92, *p* <.001.

For Test 2 (words), the model showed lower adjusted posttest reading times, relative to the no-training group, only in the phase group, *b* = −4.06, *SE* = 1.06, *t*(80) = −3.83, *p*_*corr*_ <.001.

For Test 3 (nonwords), the model showed lower adjusted posttest reading times, relative to the no-training group, only in the configuration group, *b* = −3.51, *SE* = 1.11, *t*(80) = −3.17, *p*_*corr*_ =.007.

For the DLC lexical decision test, the model showed lower adjusted posttest reading times, relative to the no-training group, only in the phase group, *b* = −8.81, *SE* = 3.59, *t*(80) = −2.45, *p*_*corr*_ =.049.

#### Reading accuracy

Regarding the model predicting the number of errors in Test 2 (words), pretest error scores significantly and positively predicted posttest error scores, *b* = 0.57, *SE* = 0.07, *t*(80) = 8.70, *p* <.001. However, the model showed no significant adjusted differences in posttest error scores between any training group and the no-training group.

#### Sensitivity analysis and Type I error rate

These analyses aimed to estimate, for each comparison between an experimental group and the control group in a linear model, the minimum effect size needed to achieve 80% power and the corresponding Type I error rate. The model compared posttest scores across groups while controlling for pretest scores.

To do this, we simulated pretest and posttest data 10,000 times for the control group and the three experimental groups. Data were generated from multivariate normal distributions chosen to reproduce the characteristics of the observed sample, except for the posttest means of the experimental groups, which were systematically varied. Each experimental group was manipulated one at a time, while the posttest means of the other groups were held constant at their reference values.

For each simulated dataset, we fitted a linear model with posttest scores as the outcome and pretest scores and group as predictors. We then extracted the p value for the contrast between the target experimental group and the control group. The proportion of *p* values below the significance threshold represented statistical power when the effect was nonzero, and the Type I error rate when the effect was zero. The significance level was set to α = 0.05/3 to account for the three planned comparisons with the control group using a Bonferroni correction.

Repeating this procedure across different posttest means yielded a power curve as a function of effect size. Effect size was expressed as the standardized coefficient beta, representing the adjusted standardized posttest difference between each experimental group and the control group after controlling for pretest scores. The full procedure was repeated separately for each experimental group and for each dependent variable (time and number of errors across the different tests). A complete description of these analyses, together with the code used, is provided on OSF (see https://doi.org/10.17605/OSF.IO/XTY4S). The results, presented in Table 6, showed that the study had enough statistical power to identify only fairly large training effects, while the Type I error rate remained below the conventional.05 alpha threshold in most cases. However, for the dependent variables Errors in Test 3 and Errors in DLC, the simulations indicated that none of the plausible posttest mean differences was sufficient to achieve adequate power.

**Table 6:** For each dependent variable and each test, the table reports the smallest mean difference (postexperimental group − postcontrol group), along with the corresponding effect size (beta), required for the lower bound of the 95% confidence interval for power to exceed 0.80

Dependent variable	Group compared with the control	Minimum mean difference	Minimum beta	Probability of Type I error [95% CI]
Time Test 2	Phase	−4.73	−0.417	0.009 [0.007, 0.011]
	Orientation	−0.88	−0.482	0.042 [0.038, 0.046]
	Configuration	0.42	−0.471	0.015 [0.013, 0.018]
Time Test 3	Phase	−3.72	−0.418	0.008 [0.006, 0.010]
	Orientation	−2.07	−0.427	0.008 [0.006, 0.010]
	Configuration	1.03	−0.534	0.088 [0.082, 0.093]
Time DLC	Phase	−9.56	−0.592	0.003 [0.002, 0.004]
	Orientation	−12.56	−0.589	0.010 [0.008, 0.011]
	Configuration	−7.06	−0.765	0.116 [0.110, 0.122]
Errors Test 2	Phase	−0.36	−0.708	0.013 [0.010, 0.015]
	Orientation	−0.49	−0.739	0.058 [0.054, 0.063]
	Configuration	−0.43	−0.685	0.010 [0.008, 0.012]
Errors Test 3	Phase	Not achieved	Not achieved	0.001 [0.000, 0.001]
	Orientation	−0.85	−0.980	0.110 [0.104, 0.116]
	Configuration	Not achieved	Not achieved	0.002 [0.001, 0.003]
Errors DLC	Phase	Not achieved	Not achieved	0.015 [0.013, 0.017]
	Orientation	Not achieved	Not achieved	0.027 [0.024, 0.030]
	Configuration	Not achieved	Not achieved	0.063 [0.058, 0.068]

The probability of a Type I error with the corresponding 95% confidence intervals is reported. As can be seen, there is not always a plausible average number of errors in the posttest that would allow for sufficient statistical power

#### Exploratory correlation between visual-search improvement and reading-speed change

As an exploratory analysis, we examined whether individual improvements in visual-search sensitivity were associated with changes in reading speed. Because *d′* was available only for the training groups, the no-training group was excluded. Visual-search improvement was indexed as *d′* Day 9 minus *d′* Day 1, whereas reading-speed change was indexed as posttest minus pretest reading time; negative values therefore indicate faster reading at posttest. Pearson correlations showed no clear evidence for a reliable association between individual gains in *d′* and changes in reading speed: Test 2, *r*(40) =.005, *p* =.973; Test 3, *r*(40) =.227, *p* =.148; and DLC, *r*(40) =.127, *p* =.424. These results, which are summarized in Table S3 in Supplementary Material 2, suggest that individual improvement in *d′* does not, by itself, explain the observed group-level transfer to reading speed.

## Discussion

This study aimed to explore a possible link between visual-search training and reading-related performance. Several findings emerged. First, *d′* values, response times, time-outs, and response criterion indicated that the three training versions differed in difficulty. The orientation version was relatively easy, the phase version was the most difficult and perceptually demanding, and the configuration version showed an intermediate level of difficulty. Across training days, participants generally improved in *d′* and became faster, suggesting task learning. However, these training changes should be interpreted as evidence of improvement within the visual-search tasks, not as direct evidence that a specific visual-search mechanism caused transfer to reading.

The regression-based analyses indicate that training-related effects were specific to reading speed and were not uniform across outcomes. The phase group showed lower adjusted posttest times for word reading and DLC, whereas the configuration group showed lower adjusted posttest times for nonword reading. These differences should be interpreted cautiously. Word reading, nonword reading, and lexical decision do not measure identical processes: word reading involves familiar lexical items, nonword reading relies more strongly on grapheme-to-phoneme conversion, and DLC performance involves silent lexical decision and visual scanning. A visual-search intervention may therefore affect some timed components of reading-related performance without producing a uniform improvement across all reading outcomes. This outcome-specific pattern argues against a broad claim that visual-search training generally improves reading ability.

The magnitude and practical significance of the effects should also be interpreted cautiously. In practical terms, the adjusted effects corresponded to approximately 4 s faster word reading for the phase group, approximately 3.5 s faster nonword reading for the configuration group, and approximately 9 s faster DLC performance for the phase group, relative to the no-training group after controlling for baseline performance. These effects suggest faster performance on timed reading-related tasks, but their clinical or educational relevance remains uncertain, especially because the sample consisted of adult typical readers. The no-training group also showed some improvement between pretest and posttest, most likely reflecting nonspecific test–retest effects such as familiarity with the testing procedure, reduced uncertainty, or practice with the assessment format. For this reason, within-group pre–post improvements cannot by themselves be interpreted as training effects; the critical comparison is the adjusted posttest difference relative to the no-training group.

Measurement reliability is another important limitation. The speed component of the DDE-2 has acceptable test–retest reliability, whereas the accuracy component is less reliable. Lower reliability increases measurement error, attenuates associations with other variables, and reduces sensitivity to training-related change. This is particularly relevant because adult typical readers made few errors, resulting in restricted variability. Therefore, the absence of consistent accuracy effects should be interpreted cautiously, and the present findings should be viewed primarily as preliminary evidence concerning timed performance rather than reliable improvements in reading accuracy.

The exploratory correlation analysis further suggests that the mechanism underlying transfer remains unclear. Individual gains in visual-search sensitivity, indexed as *d′* Day 9 minus *d′* Day 1, were not significantly associated with changes in reading speed for Test 2, Test 3, or DLC. This does not contradict the group-level adjusted posttest effects, but it indicates that transfer cannot be straightforwardly explained by a simple linear relation between individual improvement in *d′* and individual improvement in reading speed. Other factors, such as search strategy, response criterion, general visual-attentional engagement, or time-on-task, may have contributed.

Several limitations are especially relevant for interpreting the visual-search manipulation. Task difficulty was indexed by *d′*, which directly measures target–noise discriminability, and was further characterized by response times, criterion, and time-outs. However, the present design compared three visual-search tasks that differed not only in difficulty but also in target definition and likely search strategy. This differs from designs in which difficulty is manipulated within the same search paradigm, for example by parametrically varying target salience while keeping the general task structure constant. Mandal et al. (2024), for instance, manipulated visual-search difficulty by varying the tilt of a target bar among vertical nontargets, a strategy consistent with work showing that target-distractor orientation contrast modulates search efficiency (Liesefeld et al., 2016).

The three training versions may also have encouraged different search modes. According to the framework proposed by Liesefeld and Müller (2020), visual search may rely on priority guidance, when a high-priority target can be selected from a priority map, or on clump scanning, when observers inspect spatially contiguous groups of items more systematically. In the present study, the orientation condition likely encouraged priority guidance because an orthogonally oriented Gabor among homogeneous co-oriented distractors produced high target–distractor feature contrast, high *d′*, and very fast response times. By contrast, the phase condition likely required more systematic scrutiny because the target shared its orientation with the distractors and differed only in phase polarity. The configuration condition may also have required processes beyond simple priority guidance because target detection depended on the spatial arrangement of multiple Gabor elements. This interpretation remains speculative, however, because we did not manipulate set size, estimate search slopes, or record eye movements. Therefore, the observed reading-related effects cannot be attributed uniquely to task difficulty, search mode, or any specific visual-search mechanism.

A further limitation concerns training dose. Because all training versions included the same number of blocks and trials, the longer duration of the phase condition mainly reflected slower responses and more time-outs. However, equating total time-on-task in future studies would not by itself solve this problem, because it would likely result in different numbers of completed trials across conditions. Conversely, equating the number of trials can produce differences in total training duration. Future studies should therefore manipulate training dose more explicitly—for example, by including both trial-matched and time-matched control conditions, or by orthogonally manipulating task difficulty and amount of practice within the same visual-search paradigm. Future work should also manipulate difficulty within a single visual-search paradigm—for example, by varying target–distractor contrast or target salience while keeping the overall task structure constant.

Finally, the study should be considered exploratory. The sample size was constrained by practical limitations, and the sensitivity analysis indicates that the study was mainly able to detect relatively large effects. The present results therefore provide preliminary evidence that demanding visual-search training may influence some timed reading-related outcomes, but they require replication with larger samples, pre-registered analyses, and designs that separate task difficulty, search mode, and training dose.

## Conclusion

In conclusion, the present study provides preliminary evidence that a home-based visual-search training program without phonological, orthographic, or reading practice may be associated with faster performance on some timed reading-related outcomes. The effects were not general across all measures: the phase task was associated with lower adjusted posttest times for word reading and DLC, whereas the configuration task was associated with lower adjusted posttest times for nonword reading. No consistent advantage emerged for accuracy. These findings suggest a potential link between demanding visual-search practice and timed reading-related performance, but they do not yet establish a broad or clinically meaningful enhancement of reading ability. Future studies should test this possibility in larger samples, manipulate visual-search difficulty within a single paradigm, control training dose more explicitly, and evaluate whether similar effects generalize to children and to readers with dyslexia.

## Supplementary Information

Below is the link to the electronic supplementary material.

Supplementary file1 (DOCX 2861 KB)

## Data Availability

The data that support the findings of this study are available online (https://osf.io/xty4s/overview).

## Code availability

None of the code for the experiment reported here is available.
