# Supplementary material for: Probing the use of visual search as a tool to enhance reading speed
Source: Atten Percept Psychophys. 2026 Sep 23;88(7):190. doi: 10.3758/s13414-026-03336-2 (PMC13601095; doi:10.3758/s13414-026-03336-2)
Supplement: Supplementary file 1 [file 13414_2026_3336_MOESM1_ESM.docx]

Supplementary Material 2

# 1. Data loss

The training consisted of 9 consecutive days with 7 blocks of exercise performed daily. Each block involved 144 trials, for a total of 1008 trials per day. Due to technical issues in data collection using a home-based computerized training program, some trial-level data were missing or overwritten for a small number of participants. Participants nevertheless completed the planned training sessions. In these cases, d′, criterion (C), and response time indices were calculated using the available data. The missing trials were as follows:

- Orientation group: participant n. 13 had 144 missing trials on Day 1; participant n. 14 had 132 missing trials on Day 7.
- Phase group: participant n. 16 had 864 missing trials on Day 1 and 864 missing trials on Day 2.
- Configuration group: participant n. 32 had 252 missing trials on each day from Day 1 to Day 9; participant n. 34 had 144 missing trials on Day 5.

# 2. Response criterion

The response criterion indexes the participant’s general tendency to choose one of the two response options. A two-way mixed-model ANOVA showed a significant effect of Day, F(2.922, 113.953) = 44.547, p < .001, η²p = .533, indicating that criterion generally shifted from more positive/conservative values toward more negative/liberal values across training. The Group effect was significant, F(2, 39) = 8.718, p = .001, η²p = .309, and the Day × Group interaction was also significant, F(5.844, 113.953) = 11.377, p < .001, η²p = .368. The orientation group remained relatively stable, the phase group shifted from very conservative toward neutral, and the configuration group shifted from conservative toward liberal values.

Table S1. Criterion mean and standard deviation by training group.

| **Group** | **Mean criterion** | **SD** |
| --- | --- | --- |
| Orientation | 0.190 | 0.149 |
| Phase | 0.335 | 0.292 |
| Configuration | -0.137 | 0.417 |

Table S2. Criterion means across the 9 days of training.

| **Group** | **Day 1** | **Day 2** | **Day 3** | **Day 4** | **Day 5** | **Day 6** | **Day 7** | **Day 8** | **Day 9** |
| --- | --- | --- | --- | --- | --- | --- | --- | --- | --- |
| Orientation | 0.142 | 0.238 | 0.219 | 0.172 | 0.199 | 0.169 | 0.176 | 0.181 | 0.212 |
| Phase | 0.860 | 0.762 | 0.471 | 0.360 | 0.236 | 0.163 | 0.142 | 0.024 | -0.001 |
| Configuration | 0.416 | 0.249 | 0.016 | -0.066 | -0.203 | -0.327 | -0.374 | -0.484 | -0.457 |

Fig. S1. Individual daily criterion values across the three training conditions. Each line represents one participant. The orientation condition remains relatively stable, whereas the phase and configuration conditions show shifts toward less conservative response criteria across days.

# 3. Exploratory correlation between improvement in d′ and improvement in reading speed

As an exploratory analysis, we examined whether individual improvements in visual-search sensitivity were associated with changes in reading speed. Because d′ was available only for the training groups, the no-training group was excluded. Visual-search improvement was indexed as d′ Day 9 minus d′ Day 1. Reading-speed change was indexed as post-test minus pre-test reading time; therefore, negative values indicate faster reading at post-test. Pearson correlations showed no clear evidence for a reliable association between individual gains in d′ and changes in reading speed (Table S3). These results indicate that the observed group-level transfer cannot be straightforwardly explained by a simple linear association between individual gains in visual-search sensitivity and individual gains in reading speed.

Table S3. Pearson correlations between d′ improvement and reading-speed change in the training groups.

| **Reading-speed outcome** | **N** | **r** | **p** |
| --- | --- | --- | --- |
| Test 2 | 42 | 0.005 | 0.973 |
| Test 3 | 42 | 0.227 | 0.148 |
| DLC | 42 | 0.127 | 0.424 |

# 4. Additional tables and figures

Table S4. Average frequency of time-outs for each day and training group, and overall average frequency for each training group.

| **Training group** | **Day 1** | **Day 2** | **Day 3** | **Day 4** | **Day 5** | **Day 6** | **Day 7** | **Day 8** | **Day 9** | **Overall** |
| --- | --- | --- | --- | --- | --- | --- | --- | --- | --- | --- |
| Orientation | 1.21 | 0.14 | 0.29 | 0.00 | 0.00 | 0.14 | 0.07 | 0.07 | 0.07 | 0.22 |
| Phase | 46.93 | 27.36 | 22.36 | 19.07 | 17.79 | 16.64 | 20.93 | 22.14 | 15.21 | 23.16 |
| Configuration | 17.64 | 6.50 | 2.57 | 2.00 | 3.00 | 1.14 | 2.86 | 1.14 | 2.36 | 4.36 |

Table S5. Mean (SD) pre-test and post-test scores by group and dependent variable, with pre-post correlations.

| **Dependent variable** | **Group** | **Pre-test** | **Post-test** | **Pre-post correlation** |
| --- | --- | --- | --- | --- |
| Time Test 2 | Orientation | 52.62 (9.73) | 52.00 (10.23) | 0.93 |
| Time Test 2 | Phase | 47.32 (7.88) | 42.29 (5.26) | 0.87 |
| Time Test 2 | Configuration | 54.04 (8.51) | 49.49 (6.47) | 0.86 |
| Time Test 2 | No training | 48.93 (8.24) | 47.63 (7.43) | 0.91 |
| Time Test 3 | Orientation | 36.15 (7.23) | 33.16 (6.26) | 0.87 |
| Time Test 3 | Phase | 34.10 (8.04) | 31.02 (7.60) | 0.91 |
| Time Test 3 | Configuration | 40.81 (9.80) | 34.76 (7.77) | 0.75 |
| Time Test 3 | No training | 34.33 (8.83) | 33.07 (8.20) | 0.94 |
| Time DLC | Orientation | 87.71 (25.39) | 79.79 (17.96) | 0.82 |
| Time DLC | Phase | 92.71 (21.61) | 76.43 (10.85) | 0.73 |
| Time DLC | Configuration | 101.93 (34.44) | 85.14 (24.10) | 0.59 |
| Time DLC | No training | 89.61 (19.32) | 83.56 (16.17) | 0.85 |
| Errors Test 2 | Orientation | 1.00 (1.30) | 0.86 (1.41) | 0.84 |
| Errors Test 2 | Phase | 1.14 (1.35) | 0.86 (1.03) | 0.79 |
| Errors Test 2 | Configuration | 1.00 (1.30) | 0.50 (0.65) | 0.64 |
| Errors Test 2 | No training | 0.63 (0.79) | 0.53 (0.67) | 0.57 |
| Errors Test 3 | Orientation | 2.93 (1.59) | 2.86 (2.66) | 0.62 |
| Errors Test 3 | Phase | 1.57 (1.70) | 1.50 (1.45) | 0.94 |
| Errors Test 3 | Configuration | 1.79 (1.63) | 1.21 (1.37) | 0.71 |
| Errors Test 3 | No training | 1.44 (1.62) | 1.02 (1.08) | 0.35 |
| Errors DLC | Orientation | 1.00 (1.57) | 0.71 (0.61) | 0.56 |
| Errors DLC | Phase | 0.71 (1.38) | 0.64 (0.84) | 0.83 |
| Errors DLC | Configuration | 0.57 (1.16) | 0.36 (0.93) | -0.20 |
| Errors DLC | No training | 0.26 (0.73) | 0.16 (0.43) | 0.55 |

Table S6. Individual change-score summary for reading-time outcomes. Change is post-test minus pre-test; negative values indicate faster post-test performance.

| **Outcome** | **Group** | **N** | **Mean change** | **SD** | **Min** | **Max** | **Improved** | **Unchanged** | **Worsened** |
| --- | --- | --- | --- | --- | --- | --- | --- | --- | --- |
| Test 2 | Orientation | 14 | -0.62 | 3.89 | -8.50 | 7.56 | 7 | 0 | 7 |
| Test 2 | Phase | 14 | -5.03 | 4.21 | -11.27 | -0.94 | 14 | 0 | 0 |
| Test 2 | Configuration | 14 | -4.55 | 4.47 | -13.85 | 2.54 | 11 | 0 | 3 |
| Test 2 | No training | 43 | -1.30 | 3.50 | -9.85 | 8.95 | 30 | 0 | 13 |
| Test 3 | Orientation | 14 | -2.99 | 3.54 | -11.23 | 3.11 | 12 | 0 | 2 |
| Test 3 | Phase | 14 | -3.07 | 3.26 | -10.81 | 4.04 | 13 | 0 | 1 |
| Test 3 | Configuration | 14 | -6.05 | 6.44 | -25.04 | 1.15 | 13 | 0 | 1 |
| Test 3 | No training | 43 | -1.26 | 2.95 | -9.01 | 4.23 | 28 | 0 | 15 |
| DLC | Orientation | 14 | -7.93 | 14.76 | -41.00 | 15.00 | 9 | 1 | 4 |
| DLC | Phase | 14 | -16.29 | 15.59 | -61.00 | 2.00 | 12 | 1 | 1 |
| DLC | Configuration | 14 | -16.79 | 27.96 | -105.00 | 6.00 | 11 | 0 | 3 |
| DLC | No training | 43 | -6.05 | 10.08 | -35.00 | 20.00 | 35 | 1 | 7 |

Table S7. Individual change-score summary for reading-error outcomes. Change is post-test minus pre-test; negative values indicate fewer errors at post-test.

| **Outcome** | **Group** | **N** | **Mean change** | **SD** | **Min** | **Max** | **Improved** | **Unchanged** | **Worsened** |
| --- | --- | --- | --- | --- | --- | --- | --- | --- | --- |
| Test 2 | Orientation | 14 | -0.14 | 0.77 | -2.00 | 1.00 | 3 | 9 | 2 |
| Test 2 | Phase | 14 | -0.29 | 0.83 | -2.00 | 1.00 | 3 | 10 | 1 |
| Test 2 | Configuration | 14 | -0.50 | 1.02 | -3.00 | 1.00 | 5 | 8 | 1 |
| Test 2 | No training | 43 | -0.09 | 0.68 | -2.00 | 1.00 | 10 | 26 | 7 |
| Test 3 | Orientation | 14 | -0.07 | 2.09 | -4.00 | 3.00 | 5 | 5 | 4 |
| Test 3 | Phase | 14 | -0.07 | 0.62 | -1.00 | 1.00 | 3 | 9 | 2 |
| Test 3 | Configuration | 14 | -0.57 | 1.16 | -3.00 | 1.00 | 6 | 6 | 2 |
| Test 3 | No training | 43 | -0.42 | 1.61 | -5.00 | 4.00 | 20 | 14 | 9 |
| DLC | Orientation | 14 | -0.29 | 1.33 | -3.00 | 1.00 | 4 | 6 | 4 |
| DLC | Phase | 14 | -0.07 | 0.83 | -2.00 | 1.00 | 3 | 8 | 3 |
| DLC | Configuration | 14 | -0.21 | 1.63 | -4.00 | 3.00 | 4 | 8 | 2 |
| DLC | No training | 43 | -0.09 | 0.61 | -3.00 | 1.00 | 5 | 35 | 3 |

Fig. S2. Average reading time at Test 2 for each experimental condition. Error bars represent standard error; dots show individual observations.

Fig. S3. Average reading time at Test 3 for each experimental condition. Error bars represent standard error; dots show individual observations.

Fig. S4. Average reading time in the DLC lexical decision test for each experimental condition. Error bars represent standard error; dots show individual observations.

Fig. S5. Average number of errors at Test 2 for each experimental condition. Error bars represent standard error; dots show individual observations.

Fig. S6. Average number of errors at Test 3 for each experimental condition. Error bars represent standard error; dots show individual observations.

Fig. S7. Average number of errors in the DLC lexical decision test for each experimental condition. Error bars represent standard error; dots show individual observations.

Fig. S8. Individual pre-post changes in reading time for Test 2. Each grey line represents one participant; lower scores correspond to faster reading performance.

Fig. S9. Individual pre-post changes in reading time for Test 3. Each grey line represents one participant; lower scores correspond to faster reading performance.

Fig. S10. Individual pre-post changes in reading time for DLC. Each grey line represents one participant; lower scores correspond to faster performance.

Fig. S11. Individual daily d′ trajectories across the three training groups. Each line represents one participant.

Fig. S12. Individual daily response-time trajectories across the three training groups. Each line represents one participant.
